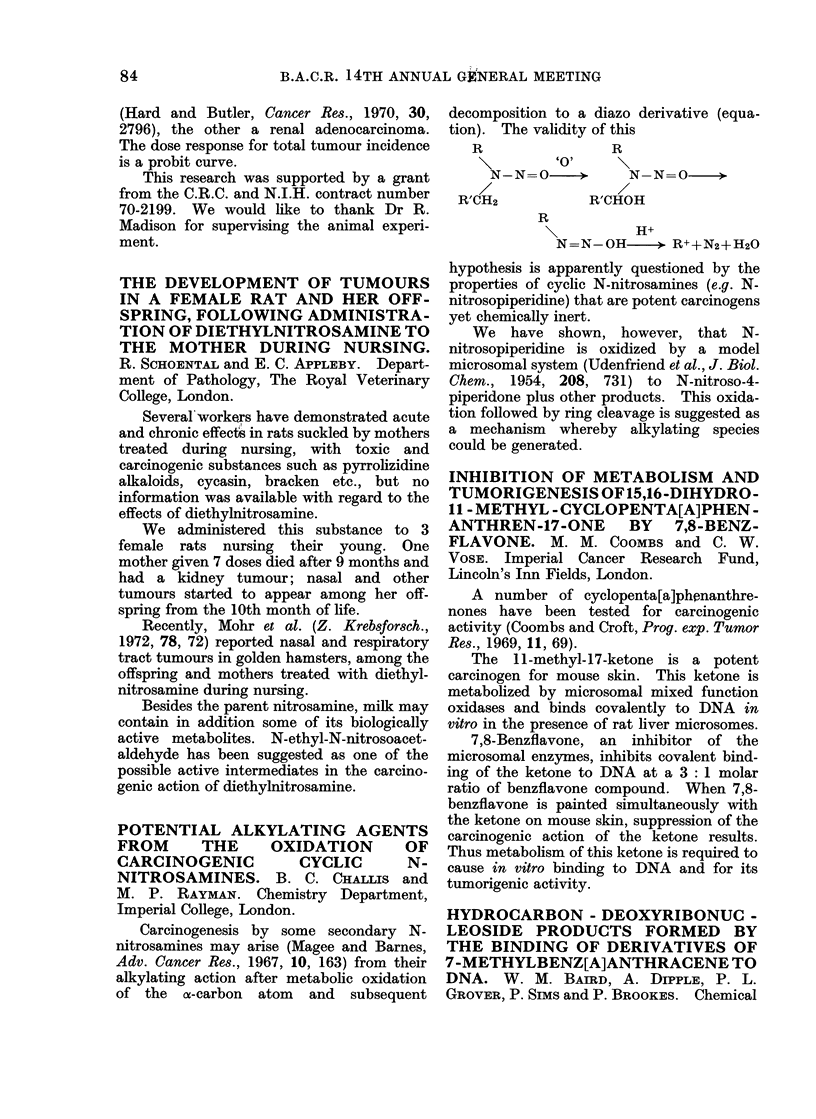# Potential alkylating agents from the oxidation of carcinogenic cyclic n-nitrosamines.

**DOI:** 10.1038/bjc.1973.101

**Published:** 1973-07

**Authors:** B. C. Challis, M. P. Rayman


### POTENTIAL ALKYLATING AGENTS FROM THE OXIDATION OF CARCINOGENIC CYCLIC N-NITROSAMINES.

B. C. Challis and M. P. Rayman. Chemistry Department, Imperial College, London.

Carcinogenesis by some secondary N-nitrosamines may arise (Magee and Barnes, *Adv. Cancer Res.*, 1967, **10**, 163) from their alkylating action after metabolic oxidation of the α-carbon atom and subsequent decomposition to a diazo derivative (equation). The validity of this hypothesis is apparently questioned by the properties of cyclic N-nitrosamines (*e.g.* N-nitrosopiperidine) that are potent carcinogens yet chemically inert.

We have shown, however, that N-nitrosopiperidine is oxidized by a model microsomal system (Udenfriend *et al.*, *J. Biol. Chem.*, 1954, **208**, 731) to N-nitroso-4-piperidone plus other products. This oxidation followed by ring cleavage is suggested as a mechanism whereby alkylating species could be generated.